# Haptic search asymmetry does not occur due to different-shaped tactile symbols on capsule paper

**DOI:** 10.1177/20416695241290466

**Published:** 2024-11-06

**Authors:** Kento Imori, Tetsuya Watanabe

**Affiliations:** 92049 Shin-etsu Chemical Co Ltd, Chiyoda-ku, Tokyo, Japan; 12978 Niigata University, Japan

**Keywords:** haptic search, search asymmetry, search time, tactile symbols, tactile maps

## Abstract

Previous research on haptic search using sandpaper with different roughness levels as a target and distractors showed that rough sandpaper among fine “pops out” and can be searched for in a shorter time than when the roles of the target and distractors are reversed. However, it is not clear whether the same search asymmetry occurs with differences in the shapes of tactile symbols on capsule paper. To explore this possibility, we conducted a haptic search experiment using circles with or without a dot on capsule paper as a target and distractors, which are often used as point symbols in tactile maps for the blind. Contrary to our expectations, haptic search asymmetry did not occur between these two tactile symbols. Regardless of target type, the search times increased in proportion to the number of items (distractors plus target), as participants tended to adopt serial search strategy in which they placed their index or middle finger on the tactile symbol to distinguish it every time they found a new one. The ratio of the search times for target-absent to target-present trials is precise alignment with the occurrence rate of repetitive search trials.

In visual search tasks, some target items are easier to find among a number of distractor items than when the roles of the target and distractor are reversed, which is a phenomenon known as search asymmetry (Treisman & Souther, 1985). For example, people can find a letter of C among a number of Os faster than they can find an O among a number of Cs. Similarly, a circle with an intersecting line among circles without a line is found faster than a circle without a line among circles with a line. Many similar experiments have been reported by Treisman and Souther (1985), Wolfe (2001), and others.

Search asymmetry has also occurred in haptic (tactile) search. For example, Plaisier et al. (2008) had blindfolded participants search with their dominant hand for stimuli consisting of circles with a diameter of 3 cm and three different levels of sandpaper roughness, and the results showed that the rough targets among fine distractors were found faster than fine targets among rough distractors. Following this experiment, they performed a series of haptic search experiments and identified haptic search asymmetry between different temperatures (Plaisier & Kappers, 2010), hardness (van Polanen et al., 2012a), and movability (van Polanen et al., 2012b).

For blind people, haptic search is a critical task utilized for reading tactile maps. In tactile maps, roads and boundaries are expressed with raised lines and traffic lights, shops, and other small landmarks are expressed with raised point symbols. Several different types of tactile point symbols are used, including basic geometric shapes (e.g., circle, triangle, square, and rectangle), hollow/filled, with/without a dot, and simple upper-case letters (e.g., C, L, T, U, V, and X) (Edman, 1992). As our team has been actively engaged in creating tactile maps for the blind and visually impaired (Watanabe & Yamaguchi, 2017), we are highly interested in the efficiency of searching tactile point symbols. Thus far, we have explored the effect of the size of tactile point symbols (Ishibashi et al., 2013) and the division of search areas (Kaga et al., 2017). If haptic search asymmetry occurs due to differences in the shapes of tactile symbols, it could be beneficial to use tactile symbols that can be found faster to express important landmarks, such as “You Are Here,” and to use less conspicuous symbols for landmarks such as traffic lights that appear in large numbers on a map. Therefore, in this work, we investigated whether search asymmetry occurs for tactile symbols having different shapes that are made on capsule paper, which is the most popular medium for tactile maps (Rowell & Ungar, 2003), by conducting a haptic search experiment. We chose circles with and without a dot as the targets and distractors, alternately, as these are the most fundamental (and thus, the most popular) tactile point symbols in tactile maps. We then evaluated haptic search asymmetry on the basis of search time, the slopes of the regression lines for the search time that is predicted to increase with the number of tactile symbols, the number of repetitive search trials, the number of errors, and search distances.

We previously reported the results of this experiment in two academic papers that did not require peer reviewing. In the first report, we described the pilot study (Imori & Watanabe, 2020), and in the second one, we reported the results from 10 participants (Imori & Watanabe, 2021). On the basis of the second report, all sections of the present paper have been written from scratch and added new results from ten more participants and on the repetitive search trials, search errors, search distances, search strategies and trajectories, and contact times analyzed carefully with videos and motion capture data.

## Experiment

### Participants and Ethics

Twenty paid graduate and undergraduate students (11 women and 9 men, ranging in age from 20 to 24 years old) participated in the experiment. None of them reported any haptic or motor dysfunction.

This experiment was reviewed by the Ethical Review Committee for Research Involving Human Subjects of Niigata University in accordance with the guidelines of the Declaration of Helsinki for research involving human subjects and was conducted with the permission of the President of Niigata University (Approval no. 2020-0161). All participants provided written informed consent.

### Stimuli

**Figure 1.**
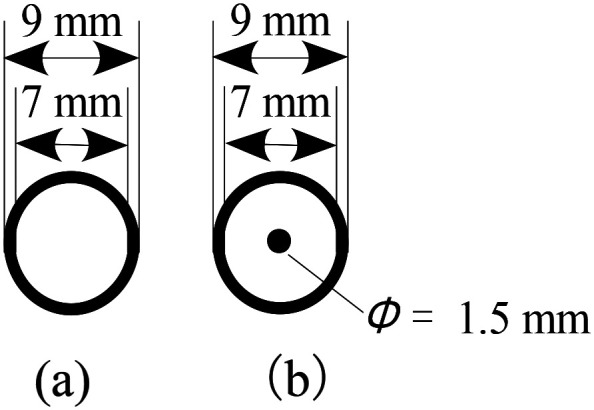
Tactile symbols. (a) Circle. (b) Circle with a dot.

The stimuli were sheets of swell paper sized 20 × 20 cm with several tactile symbols on them. Tactile symbols were circles with outer and inner diameters of 9 mm and 7 mm, respectively, drawn with a 1-mm-wide line with or without a dot with a diameter of 1.5 mm at its center (Figure 1). We refer to each of these as a “circle” and a “circle with a dot” and used them alternately as the target and distractors. We chose these circle diameters because our earlier haptic search experiment (Ishibashi et al., 2013) indicated that they could be searched the fastest.

**Figure 2.**
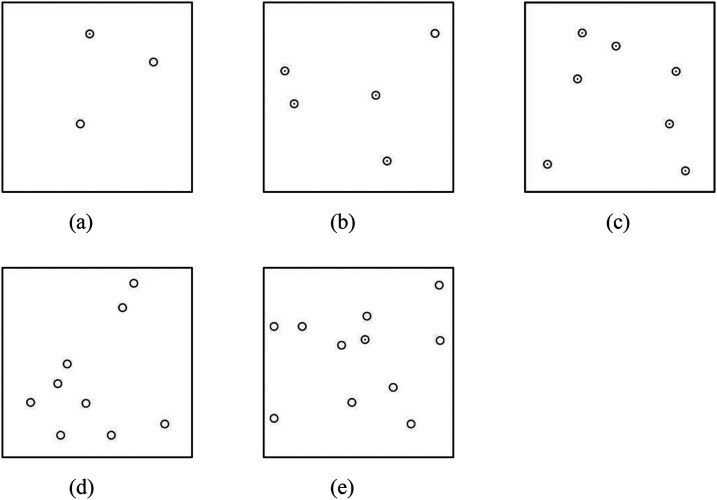
Examples of stimulus sheets with five different numbers of tactile symbols. In (a), (d), and (e), a circle with a dot is the target and the circles are the distractors, and in (b) and (c), vice versa. In (a), (b), and (e), the target is present, and in (c) and (d), the target is absent. (a) No. of symbols: 3. (b) No. of symbols: 5. (c) No. of symbols: 7. (d) No. of symbols: 9. (e) No. of symbols: 11.

The number of target and distractor items on one sheet was three, five, seven, nine, or 11. They were distributed randomly at least 1 cm away from the edges of the paper and at least 1 cm apart from each other (Figure 2). We made three sheets with different arrangements for each number of items. Two of them were used in the measurement session and the other in the training session. For each of the different numbers of items and their arrangements, four kinds of stimulus sheets were made, where the target was either a circle or a circle with a dot, and the target was either present or absent (all items were distractors). Thus, in total, we designed 5 × 3 × 4 = 60 kinds of item arrangements. Examples of stimulus sheets with five different numbers of items are shown in Figure 2.

The tactile symbols were drawn using CorelDRAW 16 (Corel) and printed in black on sheets of swell paper (ZyTex 2, Zychem) with a laser printer (LBP9510C, Canon). After passing the papers through a heating machine (PIAF, Harpo), the black part of the image swelled up to the height of approximately 0.4 to 0.5 mm (Hashimoto & Watanabe, 2016) to become tangible.

### Procedure

Participants wore an eye-mask and had infrared reflective markers attached to the fingernails of the index fingers of both hands. At the beginning of the experiment, they were given a stimulus sheet and touched the tactile symbols on it while listening to the experimenter's instructions. They were told to start searching from the bottom left of the sheet with their left hand and the bottom right with their right hand.

**Figure 3.**
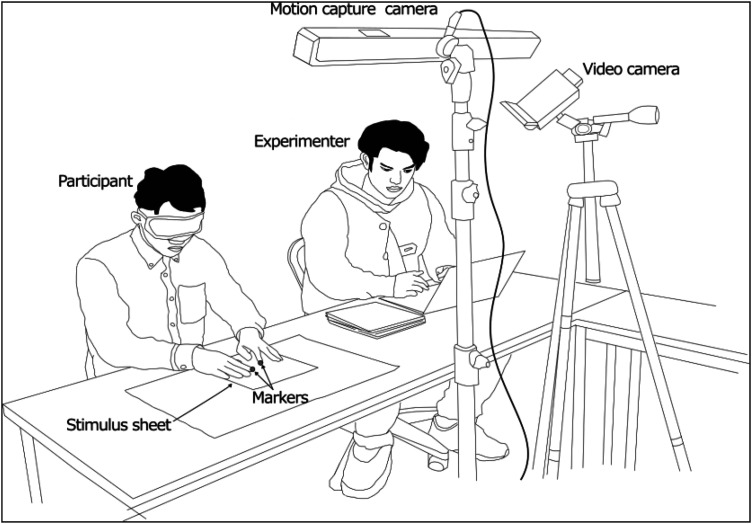
Experiment setup.

The stimulus sheets were presented on a desk in front of the participants. Four non-slip sheets and double-sided tape were utilized to prevent the stimulus sheets from moving during haptic exploration. Before each trial, the experimenter verbally indicated which was the target, a circle or a circle with a dot. Then, at the verbal cue, participants began to search for the target. As soon as they either found it or decided it was absent, they verbally answered as to the presence or absence of the target. The search time, from the experimenter's start cue to the participant's answer, was measured by a stopwatch. The participant's finger movements were recorded by a motion capture system (OptiTrack, V120:Trio), which tracked the position of the reflective markers, and a video camera (SONY, HDR-CX630 V), both of which were set up on the opposite side of the desk from the participants (Figure 3).

The experiment was divided into five blocks according to the number of items. Each block consisted of four trials of practice followed by 32 trials of the actual experiment. In half of these 32 trials, the target was a circle, and in the other half, it was a circle with a dot. In both target trials, half of the stimulus sheets had a target and the other half did not, that is, all items were distractors. The stimulus sheets were rotated by 90° and presented to the participants as different stimuli (this was not communicated to participants) to reduce the effect of the target location on the search time. The order of the 32 trials was randomized. Trials with incorrect responses (error trials) were repeated at the end of the block. All participants worked through the five blocks with a 5-min break after each block, and a total of 160 actual trials were conducted. To reduce the order effect, the order in which the different numbers of items were presented was counterbalanced across all participants.

## Results

### Search Time

**Figure 4.**
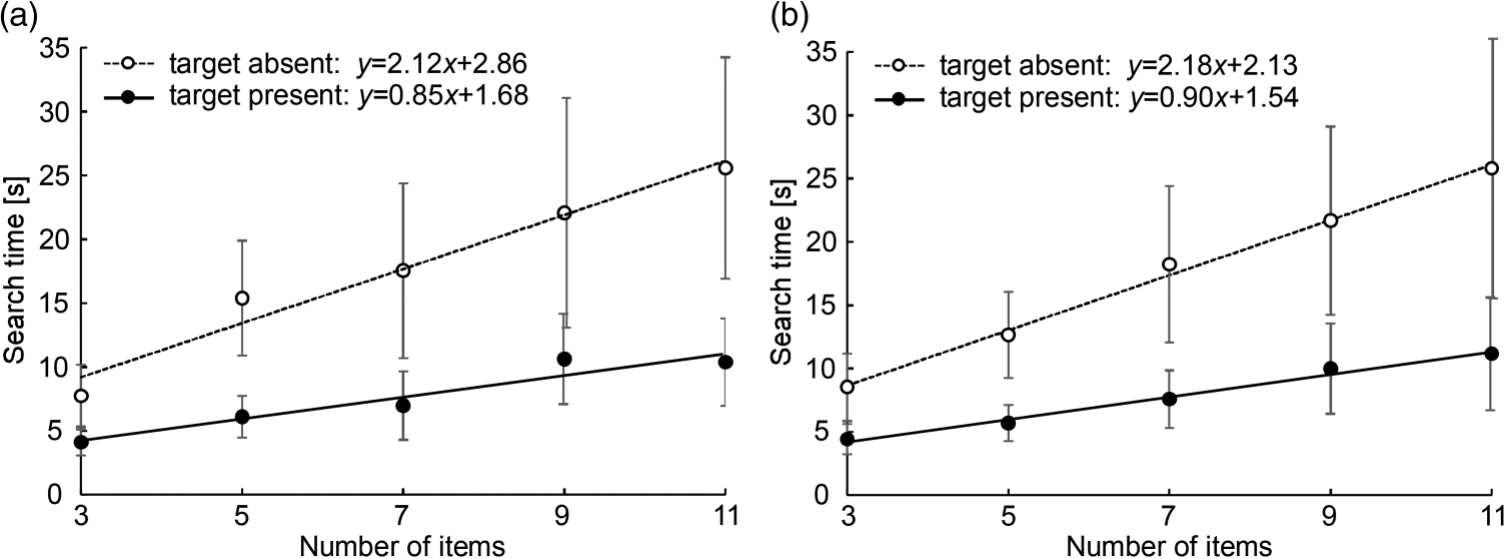
Search time and regression lines. (a) Target: circle. (b) Target: circle with a dot.

Figure 4 shows the search times averaged over all participants for each number of items and the regression lines fitted for both target-present trials (filled circles) and target-absent trials (hollow circles), where (a) shows the target of a circle and (b) shows the target of a circle with a dot. As we can see, the regression lines are well fitted for all conditions (*r *= .92, .97, .99, and .99 for target (a circle)-present, target (a circle)-absent, target (a circle with a dot)-present, and target (a circle with a dot)-absent, respectively), and their slopes are positive. This means that the search times increased in proportion to the number of items. For both target types, the search times were two times or more longer for the target-absent trials than for the target-present trials, so the slopes for target-absent trials are, accordingly, more than two times steeper than those for the target-present trials (target-present: 0.85 s/item for a circle and 0.90 s/item for a circle with a dot, and target-absent: 2.12 s/item for a circle and 2.18 s/item for a circle with a dot). This time difference is natural because the target-present trials ended as soon as the target was found whereas the target-absent trials required the participants to explore the whole sheet and, in many trials, and to repeat the exploration to make sure that the target was absent (discussed in more detail in the next section).

We calculated the slopes of the regression lines fitted for every subject in each condition and performed an analysis of variance test. The results showed a main significant effect for target presence (*F* (1, 19) = 32.6, *p *< .001), but the main effect of the target type was not significant (*F* (1, 19) = 0.07, *p *= .44). This means that no search asymmetry occurred between these two tactile symbols. Also, the interaction effect was not significant (*F* (1, 19) = 0.01, *p *= .91).

### Repetitive Search Trials

One of the main reasons for the increase in search time for target-absent trials was the increase of “repetitive search” trials, where the participants explored the whole sheet from bottom to top and then repeated the exploration. We differentiated the repetitive search trials from the nonrepetitive search trials by watching all the recorded videos.

**Figure 5.**
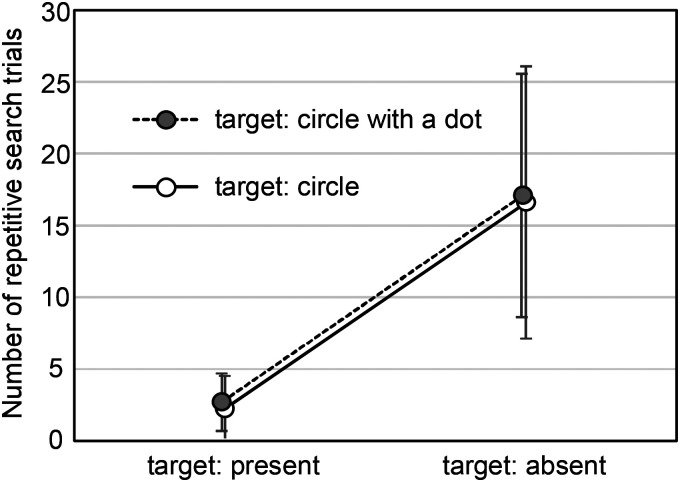
Number of repetitive search trials classified by the presence and type of the target.

Among the 3200 trials examined in total, 773 (24.1%) were identified as repetitive search trials. Of those, 674 trials occurred for the target-absent stimuli, which amounted to 42.1% of the 1600 target-absent trials, and the other 99 trials occurred for target-present stimuli, which only reached 6.2% of the 1600 target-present trials. Figure 5 shows the numbers of the repetitive search trials averaged over all participants and classified by the presence and type of the target. The total number of trials for each condition is 40, which is summed over five different numbers of items. Therefore, numbers 16 to 17 in this graph correspond to approximately 40% of the total trials for each condition. It is clear that the number of repetitive search trials differs markedly between the presence and absence of the target, and does not differ between the two target types. We analyzed the number of repetitive search trials in each condition through a variance test and found a main significant effect for target presence (*F* (1, 19) = 62.1, *p *< .001) but not for target type (*F* (1, 19) = 0.81, *p *= .38). This means that search asymmetry did not occur between these two tactile symbols even from the view of the number of repetitive search trials. Also, the interaction effect was not significant (*F* (1, 19) = 0.005, *p *= .94).

**Figure 6.**
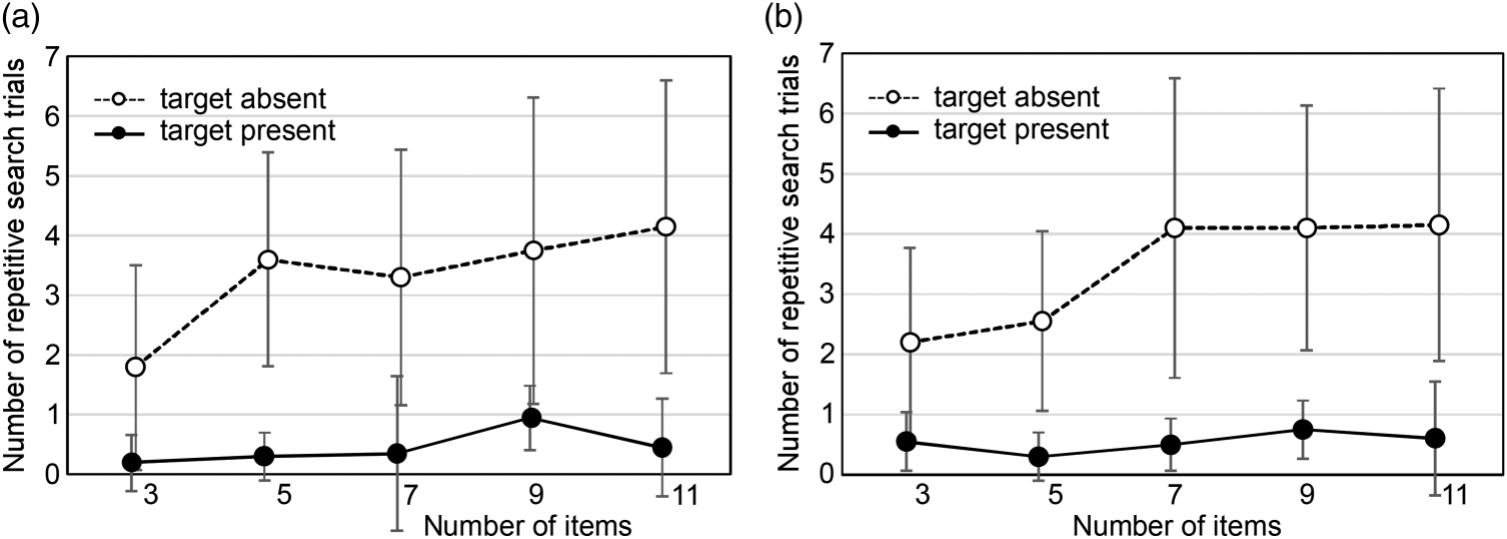
Relationship between the number of items and repetitive search trials. (a) Target: circle. (b) Target: circle with a dot.

As shown in Figure 6, the number of repetitive search trials increased when the number of items became five or more when the target was a circle and seven when the target was a circle with a dot. This suggests that the participants became less confident in counting the tactile symbols when the number of items increased to as many as five or seven.

### Search Errors

**Figure 7.**
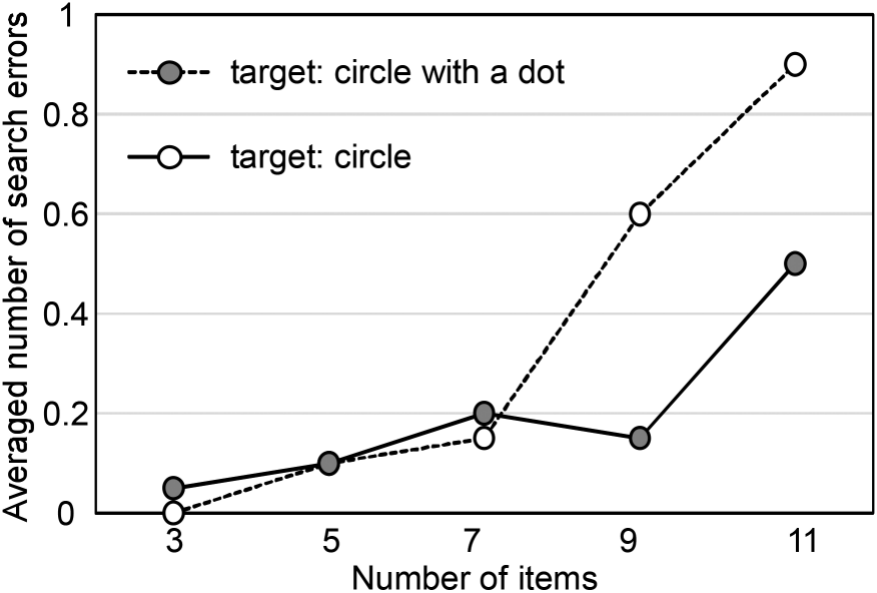
Averaged search errors for each number of items.

A total of 55 target detection errors occurred in the target-present trials, and by contrast, no errors occurred in the target-absent trials. This means that the participants never claimed to have found a target that did not exist, but sometimes missed a target that did exist. Figure 7 shows the errors averaged over all participants for each number of items. Since there were so few errors, the averaged number of errors is less than one for each number of items. For both target types, the errors increased as the number of items increased, and the number of errors for the circle target was larger than that for the circle with a dot. However, the main effect of target type was not significant (*F* (1, 19) = 3.46, *p *= .078), whereas the main effect of number of items was significant (*F* (4, 76) = 6.23, *p *< .001). The interaction effect was also significant (*F* (4, 76) = 2.91, *p *< .05).

**Figure 8.**
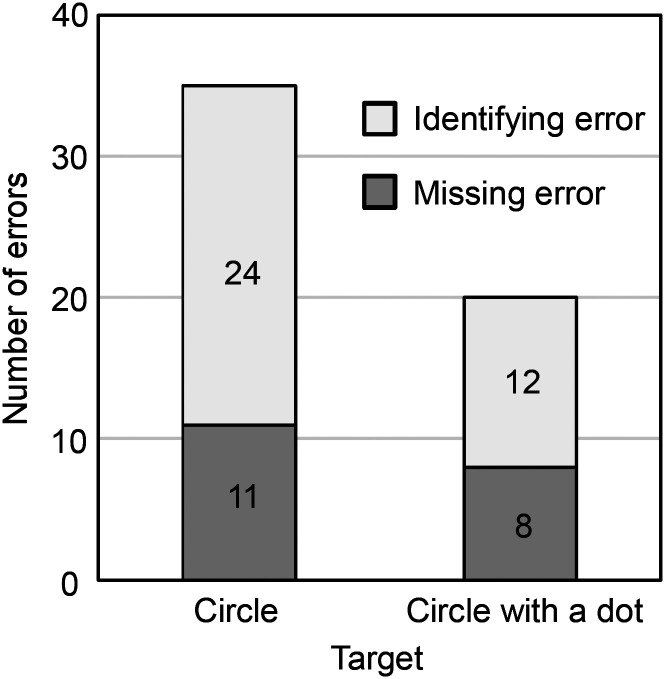
Number of missing and identifying errors for each target type.

Target detection errors can be classified into two types: identifying errors (e.g., a participant's finger passed over the target but he/she did not identify it as the target) and missing errors (e.g., a participant's finger passed by the target without touching it). We classified these two error types by watching the videos. For the circle target trials, there were 24 identifying errors and 11 missing errors (Figure 8). For the circle-with-a-dot target trials, there were 12 identifying errors and eight missing errors. These findings indicate that, in contrast to the number of missing errors, which are independent of the target type, the number of identifying errors can be an indicator of differences in the easiness of differentiating between the two target types. However, a Wilcoxon's signed rank sum test using the number of identifying errors summed over different item numbers per participant showed no significant difference between the two target types (*T *= 34, *p *> .05).

### Motion Capture Data

**Figure 9.**
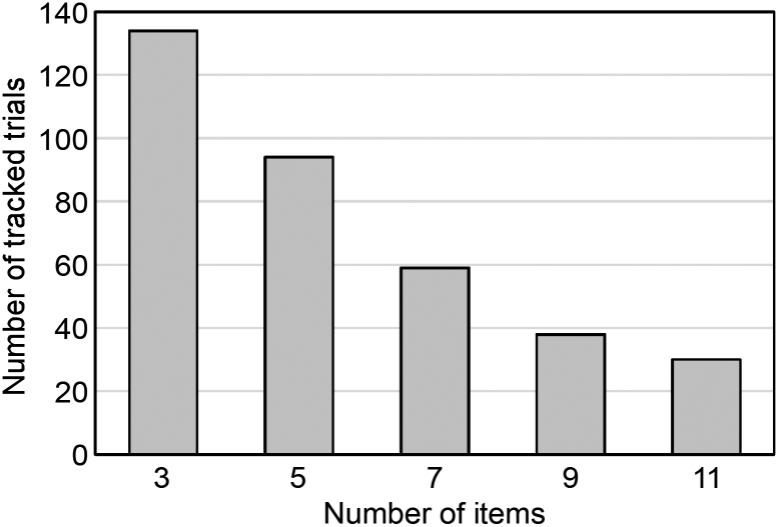
Number of tracked trials for each number of items.

The finger movement tracking often failed in the middle, maybe because of the markers being hidden from the camera or the proximity of the two index fingers. Once the tracking failed, the recovered tracking was often recorded as a different marker thereafter. Even if the number of the tracked markers was kept in two, part of data was sometimes missed. Therefore, the motion data of the two markers only with no or few missing data in the middle could be put to analysis. Furthermore, the motion data that matched with the videos were selected. As a result, the motion data of 355 trials, which is only 11.1% of 3200 trials, were retained as usable for the analysis. The number of tracked trials decreases in response to the number of items (Figure 9). This is because more items required longer search times, which increased the occurrence of tracking failures. When compared by the target type, the difference in the number of tracked trials was small; 190 trials for the circle target and 165 trials for the circle-with-a-dot target (Table 1). On the other hand, when compared to the presence of the target, the number of tracked trials without target is 59, which is about one-fifth of 296 trials with the target. This is also due to the increased frequency of tracking failures in the target-absent trials which required two times longer search times than the target-present trials.

**Table 1.** Number of tracked trials classified by the type and presence of the target.

	Circle	Circle with a dot	Sum
Absent	30	29	59
Presence	160	136	296
Sum	190	165	355

### Search Distance

**Figure 10.**
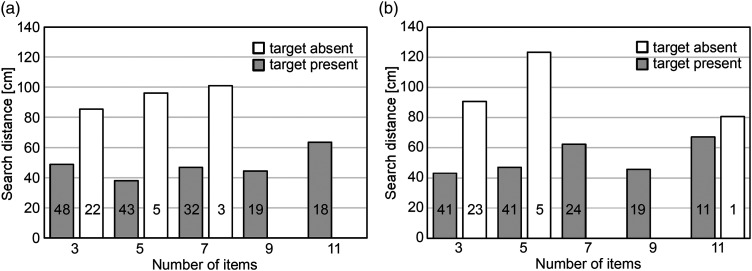
Search distances for each number of items. The numbers in the bars represent the number of tracked trials in each category. (a) Target: circle. (b) Target: circle with a dot.

The search distance of both hands were summed and averaged for each target type, presence of the target, and number of items (Figure 10). The number of trials in each category differs from 48 to one or even zero. Given that the speed of the finger movement did not differ among the conditions, the graphs of the search time (Figure 4) and search distance (Figure 10) would show the same trend. However, the search distance did not increase in proportion to the number of items. It is because the trials with longer distances are not included in this data due to tracking failures. On the other hand, it is possible to see a tendency of longer distances for the target-absent trials than the target-present trials.

### Search Strategies and Trajectories

The haptic search behaviors were observed on the video footage and outlined as follows. At the beginning of each trial, the participants placed their hands on the lower left and right edges of the stimulus sheet and began to move their hands inward and upward from there. If the symbols were not found with this first movement, then the movements to search for large areas such as wipe, zigzag, or circular motion were performed. Once the fingers contacted the tactile symbols, the fingers were moved up and down finely on the symbol to identify its type. (This movement is called “scrubbing” in Braille learning.) From time to time the fingers other than the index finger hit the tactile symbol. In that case, the participants tended to change the contacting finger to the index finger, but in some trials the first contact finger was used. If the symbol was not identified as the target, the hand was moved again to search for another symbol, or the finger stayed on the symbol so as not to lose it and the other hand was used for searching. In the trials with a high number of items, a few participants placed their multiple fingers on multiple symbols at the same time so as not to mix up the already found and still not found symbols. Most participants used both hands for searching, but a few participants sometimes searched with only one hand when the number of items was small. These movements showed a variety due to a combination of the number and location of the symbols, the location of the targets, and differences in search strategy and speed for each individual.

**Figure 11.**
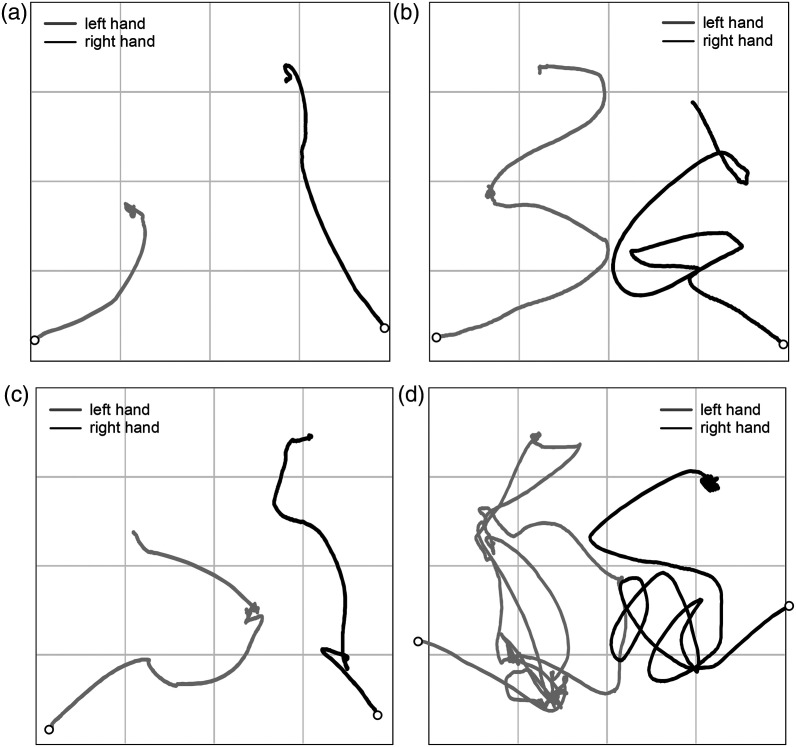
Representative search trajectories. Two circles represents the starting points. (a) Short (three items, target: circle, present). (b) Zigzag (five items, target: circle with a dot, present). (c) Unclosed polygon (seven items, target: circle with a dot, present). (d) Complex (five items, target: circle with a dot, absent).

We classified the trajectories of the finger movements from their shapes into roughly four types: short, zigzag, unclosed polygon, and complex. Figure 11 illustrates the representative trajectories of these four types. When the target was located near the starting point, the fingers hit the target symbol with the first movement and the search ended there. Thus, the trajectory became short and nearly linear (Figure 11(a)). The number of short-type trajectories is 200, which occupies 56.3% of all tracked trials, because short search trials were properly trackable. The quick and large movements to search for large areas make long curve, circular, or zigzag trajectories (Figure 11 (b)), which is 15.8% of all tracked trials. These movements tended to be made when the number of items was small. On the other hand, when the number of items was large, the fingers hit another symbol soon after they were moved. The trajectories of these trials look like an unclosed polygon, the corners of which correspond to the location of the symbols (Figure 11(c)). The ratio of these trajectories is 14.6%. In case of the stimuli with a large number of items and/or no target, the search distance became longer and the repetitive search often happened, which made the trajectories complex (Figure 11(d)). The ratio of these trajectories is 13.2%. As can be seen in Figure 11, the distinction between these four types is obscure.

### Contact Time

**Figure 12.**
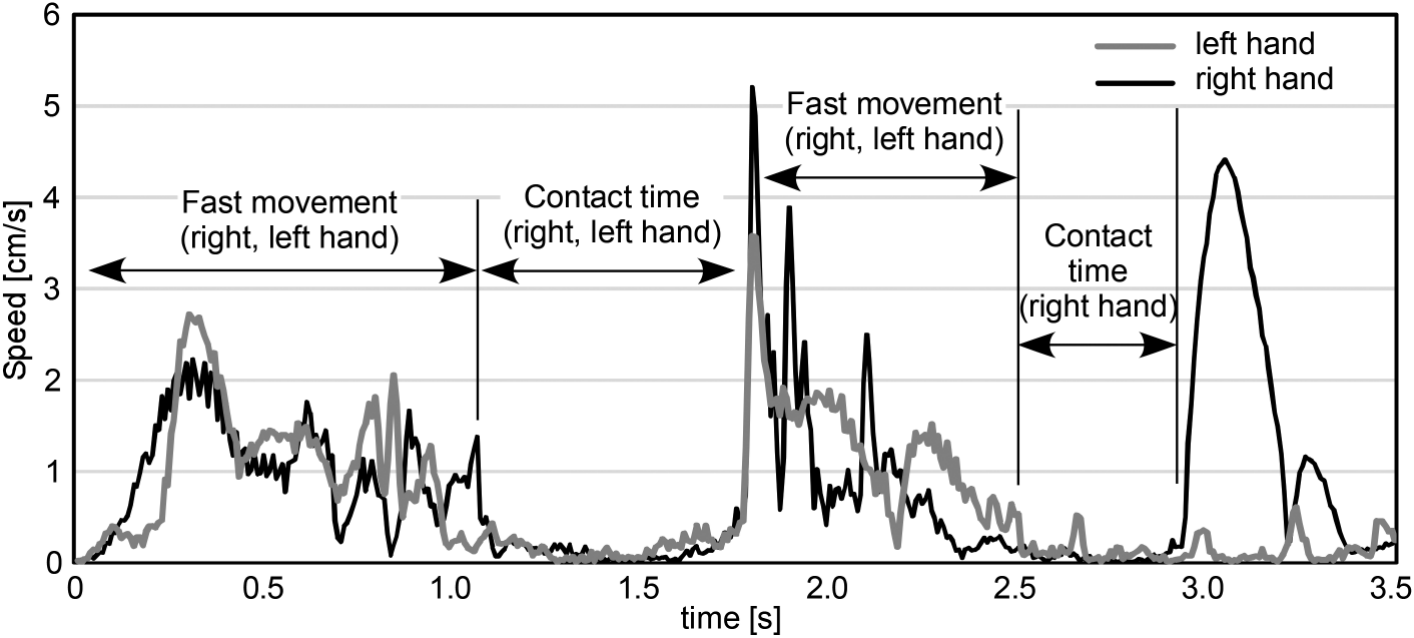
Finger movement speed. Contact times are measured as the interval between the fast movements.

The graphs of the finger movements as a function of time, i.e., speed, can visualize different finger movements: fast movements to search for the symbols, slow, pulsating movements to identify the symbol, and no movement. In Figure 12, the time durations with large movement values represent search movements and the intervals between them, symbol identification time, or contact time. The identification of contact times is, however, difficult in most of the tracked data for mainly three reasons. First, in trials where the target was found at the first movement, symbol identification movements were not followed by fast search movements and, therefore, the end of contact time was not clear. In fact, most of the trials where motion data are available were such short trials. Second, the interval between the fast movements included not only symbol identification but also finger's staying on the symbol, as described in the search strategies. Differentiating these two slow movements from the graph only is very difficult because identifying the symbol did not always accompany scrubbing. Third, the variety of the speed of large finger movements made it difficult to automatically differentiate the symbol search movements and identification movements by setting a certain threshold. Under these difficulties, we attempted to identify the contact times by matching the graphs with the videos, and as a result, only 37 contact times were identified.

**Figure 13.**
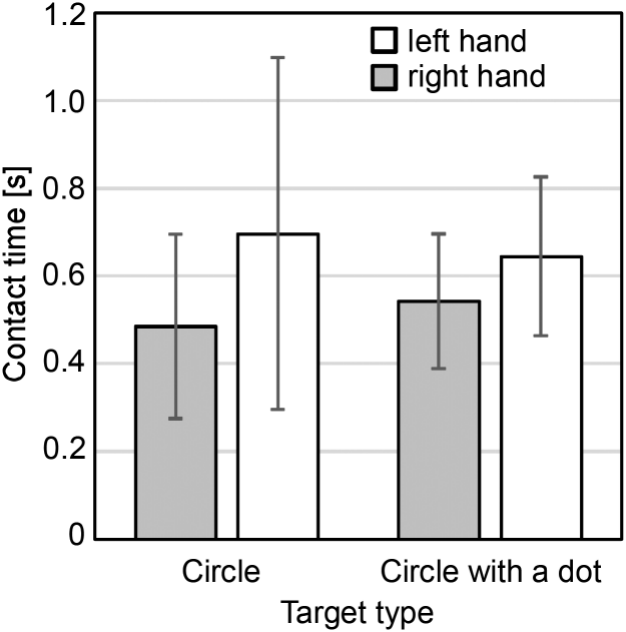
Contact time classified by the hand and symbol type.

Figure 13 shows 37 averaged contact times classified by the hand and the symbol type. The values range from 0.49 to 0.70 s. Longer times were observed for the left hand, and for circles with a dot in the case of the right hand. However, because of the small number of data and the ambiguity in measuring the start and end points of the intervals, no statistical tests were performed.

## Discussion

The search times and the slopes of their regression lines were almost the same regardless of whether the target was a circle with a dot or without a dot, that is, haptic search asymmetry did not occur. The regression lines were well fitted to the plots, which demonstrated that the search time increased in proportion to the number of items. In the prior experiment by Plaisier et al. (2008), where participants searched for circular sandpaper, the pop-out effect did not occur when searching for the fine sandpaper target among medium-rough distractors, and the slope of the regression line under this condition was 0.26 s/item for target-present trials. By contrast, in our experiment, the slopes for target-present trials were 0.85 s/item (target: circle) and 0.9 s/item (target: circle with a dot), which amounts to more than triple that of the sandpaper experiment. This suggests that the task of identifying tactile symbols on capsule paper is more difficult than discriminating the roughness of sandpaper, which is presumably why the serial search strategy was adopted. In fact, regardless of the target type it was observed that participants briefly placed their index or middle finger on the tactile symbol to identify it every time they found a new one after sweeping on the stimulus sheet. We attempted to measure the contact time that was needed to identify the symbol type by matching the trajectories and speeds of the finger movements and the videos, and found that its means ranged from 0.49 to 0.70 s. Adding the fast movement time for searching for the symbols to this contact time, it is reasonable that the search time increased to 0.85 s to 0.9 s per item.

Haptic search asymmetry did not occur in this study because the tactile difference between circles and circles with a dot is subtle. Haptically more salient symbols and textures than circles with or without a dot have been tested in many studies (for example Metzger et al., 2021; and Ryan et al., 2021 for a review), and most of these studies have used stimulus pieces as large as several centimeters or more per side. Tactile point symbols in tactile maps, however, should be as small as 1 cm to 1.5 cm in diameter, and it is desirable that they can be produced on capsule paper as it is a quick and easy production method of tactile diagrams. Overvliet et al. (2007) reported that the contact time of the finger(s) on tactile symbols created on capsule paper to find an X shape among circles was about 0.5 s. Their shorter contact times than the ones in our study suggest the possibility that the contact time required to differentiate the shapes of symbols on the capsule paper is reducible. However, the contact time is necessary in serial search, and not in parallel search, which allows discovery and identification of symbols with the whole palm of the hand.

Next to shape and texture, it is worth considering variation in elevation from the background. However, the height variation on capsule paper is as small as 0.2 to 0.3 mm (Hashimoto & Watanabe, 2016), which is not enough to perceive with the palm of the hand or the fingers. More problematic is that the change in height is largely influenced by the position of the symbols in the sheet, making it difficult to selectively elevate certain symbols.

These days, a large number of research groups have attempted to use 3D printers to create tactile maps. One of 3D printers’ advantages over capsule paper is that they can create volumetric shapes. It has been reported that this advantage led to shorter symbol search time, a high correct rate of identifying symbols, and shorter route search time (Gual et al., 2015; Brittell et al., 2018; Holloway et al., 2018; Bleau et al., 2023). By contrast, 3D printers require long hours of printing time. Thus, a technique that takes advantage of both production methods would be effective, such as using a 3D printer to print volumetric point symbols, which are then pasted on the tactile map made with capsule paper.

In a serial search task, in general, the search time for target-absent trials becomes double that for target-present trials. This is because participants in a target-present trial can end the trial as soon as they find the target and the number of stimuli to be explored is roughly half that of all the stimuli on average whereas in a target-absent trial the participants have to identify all the stimuli to make sure that no target exits. In our experiment, we observed that the participants not only explored the whole sheet but also tended to repeat the exploration. The rates of this repetitive search were 6.2% for the target-present trials and 42.1% for the target-absent trials. It is obvious that the repetitive search trials require twice as much time as that is needed to explore the whole sheet only once. Taking this time extension and the occurrence rates of the repetitive search into account, the average search time for target-absent trials was predicted to be 2.53 times that for target-present trials. The ratio calculated from our experimental results was 2.5 times when the target was a circle and 2.42 times when the target was a circle with a dot. The theoretical value of 2.53 is close to these values, and thus, we consider the way of calculating the ratio of target-absent to target-present trials in serial search to be correct.

The analysis of finger movement data gave us a lot of interesting insights into the search strategies and symbol identification. However, the scarcity of the data and their bias in number by the conditions put a limit on the analysis. We should have made sure that the finger movements could be properly tracked before conducting the experiment. Furthermore, due to a lack of synchronization between the motion capture data and video footage, it took a considerable amount of time to identify which part of the trajectory and speed data corresponded to each finger movement observed in the video, and the measured data became less reliable. The establishment of a reliable and useful measurement system will lead to new findings.
